# Poly(A)-binding protein promotes VPg-dependent translation of potyvirus through enhanced binding of phosphorylated eIFiso4F and eIFiso4F∙eIF4B

**DOI:** 10.1371/journal.pone.0300287

**Published:** 2024-05-02

**Authors:** Mateen A. Khan, Sumeyra Yumak, Hiroshi Miyoshi

**Affiliations:** 1 Department of Life Sciences, College of Science and General Studies, Alfaisal University Riyadh, Riyadh, Saudi Arabia; 2 Department of Science, Borough of Manhattan Community College, City University of New York, New York, NY, United States of America; 3 Department of Microbiology, St. Marianna University School of Medicine, Kawasaki, Japan; Ohio State University, UNITED STATES

## Abstract

The phosphorylation of eukaryotic translational initiation factors has been shown to play a significant role in controlling the synthesis of protein. Viral infection, environmental stress, and growth circumstances cause phosphorylation or dephosphorylation of plant initiation factors. Our findings indicate that casein kinase 2 can phosphorylate recombinant wheat eIFiso4E and eIFiso4G generated from *E*. *coli in vitro*. For wheat eIFiso4E, Ser-207 was found to be the *in vitro* phosphorylation site. eIFiso4E lacks an amino acid that can be phosphorylated at the position corresponding to Ser-209, the phosphorylation site in mammalian eIF4E, yet phosphorylation of eIFiso4E has effects on VPg binding affinity that are similar to those of phosphorylation of mammalian eIF4E. The addition of VPg and phosphorylated eIFiso4F to depleted wheat germ extract (WGE) leads to enhancement of translation of both uncapped and capped viral mRNA. The addition of PABP together with eIFiso4Fp and eIF4B to depleted WGE increases both uncapped and capped mRNA translation. However, it exhibits a translational advantage specifically for uncapped mRNA, implying that the phosphorylation of eIFiso4F hinders cap binding while promoting VPg binding, thereby facilitating uncapped translation. These findings indicate TEV virus mediates VPg-dependent translation by engaging a mechanism entailing phosphorylated eIFiso4Fp and PABP. To elucidate the molecular mechanisms underlying these observed effects, we studied the impact of PABP and/or eIF4B on the binding of VPg with eIFiso4Fp. The inclusion of PABP and eIF4B with eIFiso4Fp resulted in about 2-fold increase in affinity for VPg (*K*_d_ = 24 ± 1.7 nM), as compared to the affinity of eIFiso4Fp alone (*K*_d_ = 41.0 ± 3.1 nM). The interactions between VPg and eIFiso4Fp were determined to be both enthalpically and entropically favorable, with the enthalpic contribution accounting for 76–97% of the ΔG at 25°C, indicating a substantial role of hydrogen bonding in enhancing the stability of the complex. The binding of PABP to eIFiso4Fp·4B resulted in a conformational alteration, leading to a significant enhancement in the binding affinity to VPg. These observations suggest PABP enhances the affinity between eIFiso4Fp and VPg, leading to an overall conformational change that provides a stable platform for efficient viral translation.

## Introduction

Viruses have paved the way for the understanding of many aspects of host cell machinery which is crucial for translation. One of the primary events in translation is the engagement of mRNAs by the eukaryotic translation initiation factor eIF4F. eIF4E recognizes the cap structure (m7GpppN, where N is any nucleotide) located at the 5’-end of mRNAs, while both the poly(A)-binding protein (PABP) and the eIF4G interact with the poly(A)-tail. Similarly, eIFiso4F, an isoform of eIF4F found in higher plants [1, 2], comprises of eIFiso4E, eIFiso4G, and eIF4A which engage in analogous interactions as the eIF4F subunits. Both eIF4F and eIFiso4F play a crucial role in facilitating cap-dependent *in vitro* translation [1]. Both eIF4F and eIFiso4F, the two plant proteins, share multiple functional similarities. They can support translation *in vitro*, facilitating ATP-dependent helicase activity, and serving as RNA-dependent ATPases [2–5]. eIF4F, as does the plant specific eIFiso4F, plays a pivotal role in recruiting various initiation factors, including eIF4A, which serves a dual function of simultaneously tethering mRNAs to ribosomes and facilitating the search for the translation start site within the mRNA sequence. The interaction between PABP and eIF4F leads to a functional outcome wherein PABP shows an increased affinity for the poly(A)-tail, while eIF4F demonstrates an enhanced affinity for the 5’-cap structure [6]. Association between PABP and eIF4F leads to mRNA circularization, a process that facilitates multiple successive rounds of translation [7]. eIF4F was observed to exhibit superior translation support for RNA molecules containing secondary structure in the noncoding region compared to eIFiso4F [8]. The findings from oligonucleotide binding studies reveal that while eIF4F’s binding affinity is notably influenced by the presence of secondary structure, eIFiso4F, in contrast, exhibits a discernible preference for linear structures during the binding process [9]. Moreover, studies showed that *in vitro* translation of the *tobacco etch virus* (TEV) eIFiso4G does not have role in cap-independent translation while eIF4G does [10]. Binding studies demonstrated a correlation between the binding affinity of eIF4G and eIFiso4G and the efficiency of translation [11]. The interaction between PABP and eIF4G augments the poly(A) binding activity by enhancing the binding of eIF4F with the cap structure and diminishing its dissociation rates [12, 13]. The binding of eIF4B and eIF4G not only enhances the affinity of PABP for poly(A)-RNA but also synergistically impacts PABP activity [14], indicating that their physical binding promotes the formation of a stable complex, thereby enhancing their functional role in translation initiation.

Potyvirus infection of crops has devastating economic consequences [15]. They are members of the picornavirus of positive-stranded RNA viruses. A diverse range of translation initiation mechanisms has been adopted by viruses. While a significant number of viruses employ a cap-dependent mechanism, some viruses rely on cap-independent mechanisms for their translation initiation. In the potyvirus viral RNA genome, the 5’-terminus is not capped; rather, it forms a covalent linkage with a viral protein genome linked, VPg, through a tyrosine residue [16]. Potyviruses exhibit the capability of cap-independent translation [16], underscoring the pivotal role of VPg in facilitating the translation process. The infectivity of viral RNA is reliant on the covalent linkage between VPg and viral RNA, mediated by a tyrosine residue, with studies demonstrating its necessity for infectivity [17], and *in vitro* degradation of VPg influencing the level of infectivity of released viral RNA during virion disassembly [18]. VPg, a key component of potyvirus, assumes critical functions throughout the viral infection cycle, encompassing crucial roles in cell-to-cell movement, viral replication, and long-distance movement [17]. VPg is believed to participate in polyprotein translation by interacting with cap-binding proteins such as eIF4E, eIFiso4E, and other initiation factors, as suggested for numerous viruses [18–20]. The translation initiation of viral RNA is mediated by VPg through its protein-protein interactions with the translation initiation machinery. The interaction between the VPg protein of turnip mosaic virus and eIF4E has been identified as a crucial factor influencing the infectivity of the virus [21].

VPg of potyvirus such as *Turnip mosaic virus* (TuMV) and *Tobacco etch virus* (TEV) interacts with eIF4E and eIFiso4E of wheat germ [19, 22]. The connection between plant RNA virus infection and translation initiation factors has been established in previous studies [23], highlighting its significance. These observations indicate the significance of VPg in protein synthesis initiation, potentially acting as a cap analogue [24]. Earlier studies [19, 25] have demonstrated robust interactions between VPg and plant eIFiso4E or eIF4E, indicating a potential competitive binding scenario where VPg can effectively compete with cap binding. According to reports, VPg forms the eIFiso4F∙VPg∙IRES complex through preferential interactions with eIFiso4E and eIFiso4F, which in turn enhances uncapped viral translation [18, 26]. Furthermore, quantitative binding studies [19] showed that VPg preferentially connects with eIFiso4F as opposed to eIF4F, and that eIFiso4F’s affinity for VPg rises even more upon the inclusion of eIF4B and PABP [27]. Phosphorylation of eIFiso4E (Ser-207) by casein kinase 2 resulted in an enhanced binding affinity and increased translational efficiency with VPg [23, 28]. All eukaryotic cells include the cytoplasm and nucleus of protein kinase CK2, a widely distributed and highly conserved serine/threonine kinase [29]. It has been demonstrated that plant casein kinase II phosphorylates several initiation factors, such as eIF4B, eIF2α, eIF2β, eIF3c, and eIF5 [30]. Ser-207 has been identified as the phosphorylation site for eIFiso4E. Reports of the phosphorylation of wheat eIFiso4E, eIFiso4G, and eIF4B in vitro have been made [31]. It is currently unknown how wheat eIFiso4E and eIFiso4G control translation in vivo and get phosphorylated. Phosphorylation of plant initiation factors is induced by various stimuli such as stress, heat shock, oxygen deprivation, developmental cues, and the photosynthesis rate [32, 33]. Phosphorylation of mammalian eIF4E leads to increased affinity for cap mRNA, resulting in enhanced translation efficiency [34]. Phosphorylation-mediated manipulation of the host cell translation machinery is a strategy employed by viruses. Previous studies [28, 29] have demonstrated that phosphorylation enhances the binding affinity and translation efficiency of eIFiso4E with VPg. However, whether PABP affects the binding affinity and translation efficiency of phosphorylated eIFiso4F with VPg as it does for m7G cap and eIFiso4F has not been elucidated. To examine the role of VPg in viral translation initiation under phosphorylation conditions, we further examined the effect of PABP and eIF4B on the translation initiation and equilibrium binding of VPg with phosphorylated eIFiso4F (eIFiso4Fp). The addition of PABP increased the protein synthesis and binding affinity of eIFiso4Fp, eIFiso4Fp∙4B with VPg. Thermodynamic analyses demonstrated that the binding of eIFiso4Fp·4B·PABP to VPg resulted in an increase in enthalpic contribution and a decrease in entropic contribution, indicating enhanced hydrogen bonding and an overall conformational change.

## Materials and methods

### Purification of initiation factors

cDNAs for the wheat translation initiation factors (eIFiso4E, eIFiso4G, eIF4B) were obtained from a wheat cDNA library prepared in λgt11 [35] and was the source for the cDNAs used to prepare the plasmids for expression in *E*. *coli*. Wheat eIFiso4E and eIFiso4G recombinant proteins were expressed in BL21 (DE3) pLysS *E*. *coli* containing pET3d plasmid as described previously [36, 37]. Isolation of eIFiso4E was accomplished using the HiTrap Mono-Q column and m7GTP-Sepharose affinity columns (GE Healthcare). The protein bound to the affinity column was effectively released by elution with 100 mM GTP. The collected fractions were subjected to 10% sodium dodecyl sulfate-polyacrylamide gel electrophoresis (SDS-PAGE) for analysis. The eIFiso4G protein was isolated using a HiTrap SP column. The elution of the recombinant eIFiso4G protein was achieved using a linear gradient of KCl (50–500 mM). eIFiso4G appeared in the fractions of 200–300 mM KCl. The pooled purified fractions were concentrated using a Centricon-10 micro concentrator. The protein’s purity was confirmed through 10% SDS-PAGE analysis.

Recombinant wheat eIF4B and PABP clones were generous gifts from Prof. Daniel Gallie (University of California, Riverside, CA, USA). The recombinant proteins were His-tagged and were purified from bacterial cultures using His Trap HP columns (GE Healthcare). eIF4B was purified from *E*. *coli* containing the constructed pET3d vector in BL21 (DE3) pLysS extracts containing the expressed protein as described elsewhere [36, 38]. Wheat cDNA expression library encoding for PABP was used for *E*. *coli* expression constructs [39]. Wheat PABP expression was achieved in *E*. *coli* using the pET19b vector in BL21 (DE3) pLysS strain, and purified as previously described method [40]. Cells were expressed in a liquid broth medium at 37°C with the addition of ampicillin (100 μg/ml) and chloramphenicol (34 μg/ml). Cells were induced with isopropyl-1-thio-β-D-galactopyranoside (0.1g/L) for 5h, followed by harvesting the cells by centrifugation. Pellets were suspended in 20 mM Tris-HCl, pH 7.9 binding buffer containing 500 mM NaCl, 5 mM imidazole, 0.5 ml of aprotinin, 1.0 mM phenylmethylsulphonyl fluoride and trypsin inhibitor (100 μg/ml) at 4°C. The lysate was subjected to sonication to disrupt the cells, followed by centrifugation to separate the supernatant from the precipitate. The supernatant was loaded into a His-Tag nickel column. The binding buffer was used to wash the column, and subsequently, the bound protein was eluted using an elution buffer consisting of 20 mM Tris-HCl, pH 7.9, 5 mM NaCl, and 200 mM imidazole. The protein that was separated was subjected to dialysis against a buffer consisting of 20 mM Tris-HCl, pH 7.6, with 100 mM NaCl and 5% glycerol. The confirmation of the purity of eIF4B and PABP was accomplished through 12% SDS-PAGE, followed by staining with Coomassie brilliant blue. Preceding the spectroscopy measurements, the protein samples underwent dialysis using a titration buffer (composed of 20 mM Tris-HCl, pH 7.6, containing 100 mM NaCl, 1 mM MgCl_2_, and 1.0 mM DTT). The protein concentrations were quantified using a Bradford assay, employing bovine serum albumin as the standard [41].

### Preparation of potyvirus VPg

VPg from turnip mosaic virus was expressed and purified using the established protocol described elsewhere [25]. The cDNA clone of TuMV VPg plasmid was constructed from the pET-21a and the resulting construct was subjected to digestion with NdeI and XhoI restriction enzymes. His-tagged VPg was expressed [25] in BL21 (DE3) pLysS *E*. *coli* cells. The growth of cells took place in Luria-Bertani (LB) medium at a temperature of 37°C. Following induction with 100 μM isopropyl 1-thio-β-D-galactopyranoside (IPTG) for 6 hours at 37°C, the cells were harvested, the cell pellets were resuspended in a 20 mM Tris-HCl, pH 7.5 buffer containing 100 mM NaCl at 4°C, and cell disruption was achieved through sonication. The supernatant was passed through a Ni-Sepharose column (Novagen), and the protein bound to the column was eluted with a 20 mM Tris-HCl, pH 8.0 buffer containing 100 mM NaCl and 250 mM imidazole. The sample was subjected to dialysis using a 20 mM Tris-HCl, pH 8.0 buffer supplemented with 1 mM DTT and 5% glycerol. The protein sample was subsequently loaded onto a HiTrap-Mono-Q anion exchange column. The sample was subjected to dialysis against a 20 mM Tris-HCl, pH 7.6 buffer supplemented with 50 mM NaCl, and 5% glycerol. The purity of the VPg protein was subsequently verified through 15% SDS-PAGE analysis. All samples were dialyzed against a 20 mM Tris-HCl, pH 7.6 buffer containing 100 mM NaCl, 1 mM MgCl_2_, 1 mM DTT, and 0.1 mM EDTA, and subsequently subjected to filtration through a 0.22 μM Millipore filter prior to the spectroscopy measurements. The protein concentrations were quantified using a Bio-Rad protein assay reagent (Bio-Rad Laboratories, CA) and the Bradford assay, employing bovine serum albumin as the standard [35].

### Phosphorylation of initiation factor eIFiso4F

The *in vitro* phosphorylation of recombinant eIFiso4E and eIFiso4G were conducted using casein kinase II (CK2) following the previously described protocol [31]. The cloning and expression of wheat CK2 following previously described protocol [30, 42]. A reaction mixture consisting of 500 μg of eIFiso4F, 50 units of casein kinase, and 200 μM ATP, 1 mM MgCl_2_, 100 mM NaCl, 1 mM DTT, and 0.1 mM EDTA in a 0.5 ml volume of 20 mM Tris-HCl, pH 7.0, buffer was incubated at room temperature (25°C) with continuous rotation for 3 hours. The phosphorylated initiation factor sample was incubated at room temperature with m^7^-GTP-sepharose beads for 2h with rotation. The casein kinases were removed by spinning down the Sepharose and followed by careful removal of the supernatant. The bound phosphorylated initiation factor iso4F to m^7^-GTP-sepharose was eluted as described above. Then the mixture was passed through a desalting column to separate ATP from the sample, and transfer samples into a titration buffer for spectroscopic measurements. The initiation factor iso4F phosphorylation efficiency was measured by nondenaturing gel electrophoresis. The degree of initiation factor phosphorylation was determined by Mass spectroscopic analysis as described previously [31].

### Translation assay

TEV_1-143_-*luc*-A_50_ mRNA transcript that contains TEV leader sequence and terminates in a poly(A)_50_ tract was translated in non-depleted WGE and depleted WGE as described previously [43]. Wheat germ lysate was isolated following the previously described method [25]. The depleted WGE was prepared following the Promega instructions manual. A 200 μl aliquot of WGE was mixed with 300 μl of m^7^GTP-sepharose or poly(A)-agarose column and the sample mixture was incubated with continuous mixing at 5°C for 30 min. The lysate was obtained through high-speed centrifugation. The resin was resuspended in buffer containing 100 mM GTP. The GTP eluted fractions of eIFiso4F (eIFiso4E, eIFiso4G), eIF4B, and PABP from WGE was confirmed by running western blot analysis following resolution of the lysate by 12% sodium dodecyl sulfate-polyacrylamide gel electrophoresis as shown previously [10, 44]. TEV mRNA (1.0 μg) construct was translated using non-depleted or depleted WGE for luciferase activity in translation assay 20 mM Tricine pH 8.0 buffer containing 1 mM MgCl_2_, 0.1 mM EDTA, 100 mM NaCl, 1mM DTT, 0.25 mM coenzyme A, 500 μM ATP, 50 Units of RNase inhibitor and 10 μM of complete amino acid mixture (Promega). Supplementation of depleted wheat germ lysates involved the addition of phosphorylated eIFiso4F, eIF4B, and PABP purified either from recombinant initiation factors, as indicated in each experiment. VPg protein (20 nM) was added to the translation mixture in non-depleted and depleted WGE. After incubating the reaction mixtures at 25°C for 90 minutes, 0.5 μl aliquots were subjected to luciferase activity assays, and the emitted light was measured following the addition of 500 μM luciferin. TEV_1-143_ mRNA nucleotide sequence was synthesized *in vitro* using Promega RiboMAX^TM^ SP6 RNA production system following the Promega RiboMAX^TM^ instruction manual. The synthesis of capped mRNA involved utilizing 5 μg of template in a reaction mixture similar to that of uncapped mRNA synthesis, with the addition of 1 mM GTP and 25 mM m7GpppG. With the provided conditions, over 95% of the mRNA underwent capping, and the concentration of RNA was assessed by measuring the optical density at 260 nm. The purity of the synthesized RNA was validated through 1% agarose gel electrophoresis and determination of the absorbance ratio (A_260_/A_280_ nm) in diethylpyrocarbonate-treated water.

### Fluorescence binding analysis

Fluorescence intensities were recorded on a spectrofluorometer (Shimadzu, RF-6000), using a quartz cuvette with a path length of 10 mm and excitation and emission slits set at 4 nm and 5 nm, respectively. The sample was thermostatic at the temperature of 25 ± 0.2°C using a built-in a thermocouple within the cuvette for all samples unless specified for temperature dependence studies. The fluorescence intensity of each sample was measured by excitation at 280 nm and the emission was measured at 334 nm. Direct fluorescence titration was used to study eIFiso4Fp•VPg and eIFiso4F•VPg interactions. A 500 nM aliquot of eIFiso4F, eIFiso4Fp, eIF4B, PABP, eIFiso4Fp•4B, eIFiso4Fp•PABP, and eIFiso4Fp•4B•PABP were incubated with increasing concentrations of VPg in 0.5 ml of titration buffer 20 mM HEPES/KOH, pH 7.6 buffer containing 100 mM NaCl, 1 mM MgCl_2_ and 1 mM DTT. The interaction between VPg and initiation factors was assessed by monitoring the decrease in fluorescence intensity of the eIFs. The fluorescence intensity of each sample (eIFs•VPg complex) was obtained by subtracting the fluorescence intensity of each separate sample with each concentration of VPg. The corrected fluorescence was used to measure the dissociation constant of the complex. When necessary, fluorescence intensities were adjusted for dilution and corrected for the inner filter effect. The normalized (ΔF/ΔF_max_) fluorescence difference between the eIFs-VPg complex and the sum of the individual fluorescence intensity were used to determine the equilibrium binding affinity (*K*_a_) or dissociation constant (*K*_d_). ΔF_max_ is the fluorescence change for complete saturation of eIFs with VPg. To find the value of ΔDFmax from the intercept, the linear double reciprocal plot of 1/ΔDF vs 1/[VPg] is extrapolated to the ordinate [11, 45]. A comprehensive description of the data fitting procedures can be found elsewhere [46, 47]. Kaleida Graph software (version 2.1.3; Abelbeck Software) was used to perform nonlinear least squares fitting of the data. The average of data values from three independent titration experiments was utilized for calculations. Previously reported [14, 27, 48] *K*_d_ values for the interaction of eIFiso4F•4B, eIFiso4F•PABP, and eIFiso4F•4B•PABP were employed to determine the concentrations that had more than 90% of the eIFiso4F in the complex form at the lowest protein titration point of 50 nM. The molar ratio of eIFiso4Fp to eIF4B or PABP in the binding solution was 1:10. The molar ratio of the eIFiso4Fp•4B•PABP complex was 1:10:30. A 15-minute incubation period was employed to ensure the formation of protein complexes before data collection [27, 49]. Three separate titration experiments were carried out for each equilibrium measurement, and the average result is shown.

### Thermodynamic analysis

Temperature dependence of the equilibrium binding constant was employed to determine the thermodynamic parameters for eIFiso4Fp, eIFiso4Fp•4B, eIFiso4Fp•PABP, and eIFiso4Fp•4B•PABP complex to VPg. Enthalpy (ΔH), entropy (ΔS), and free energy (ΔG) were determined by analyzing the Van’t Hoff plots of ln *K*_eq_ *versus* 1/T, employing the following equations for calculations:

$$-RT\;ln\;K_{eq}=\Delta H-T\Delta S$$ (Eq 1)

$$\Delta G=-RT\;ln\;K_{eq}$$ (Eq 2)

where R represents the universal gas constant, and T denotes the absolute temperature. *K*_eq_ represents the equilibrium association constant at various temperatures. ΔH and ΔS were determined by analyzing the slope and intercept, respectively, of the ln *K*_eq_ *versus* T^-1^ (Kelvin) plot. The titration reactions were thermostatic at 5, 10, 15, 20, and 25°C. The titrations were conducted following the previously described procedure. Temperature monitoring inside the cuvette was achieved by employing a thermocouple. The change in free energy (ΔG) of the initiation factors complexes with VPg was calculated at 25°C from Eq (2).

### Circular dichroism measurements

Circular dichroism (CD) spectra were measured on a Chirascan Plus spectropolarimeter (Applied Photophysics Ltd, UK). Far-UV CD spectra were obtained from 200–260 nm at 25 ± 0.2°C, controlled thermostatically with a circulating water bath connected with a cell holder at constant nitrogen flow. Phosphorylated eIFiso4F were obtained in titration buffer 20 mM Tris-HCl, pH 7.6, 100 mM NaCl, 1 mM MgCl_2_, and 1 mM DTT, with a 0.5 mm path-length quartz cell. Calibration of the instrument was conducted using (D)-(+)-10-camphorsulfonic acid following the manufacturer’s guidelines. Initiation factors complex formation was performed as described previously. To keep the same salt concentration in a reaction mixture, all the proteins were dialyzed against the same titration buffer containing same salt concentrations before adding to binding buffer that contained the same salt concentration as the VPg and eIFs solutions. VPg and eIFs mixtures were incubated in titration buffer. Before scanning the data, each sample of eIFiso4Fp, eIFiso4Fp∙eIF4B, eIFiso4Fp∙eIF4B∙PABP complexes in the absence and presence of VPg were pre-incubated for 15 minutes at 25°C to form the protein-protein complex. The far-UV CD spectra of eIFiso4Fp (0.1 μM) with VPg (1 μM) were obtained in the presence of eIF4B (1 μM) and PABP (10 μM). Each spectrum was recorded at a scanning rate of 50 nm/min with a response time of 1 second. Each spectrum was obtained by averaging three scans. High-frequency noise reduction was implemented for all spectra prior to the final measurements of CD spectra. The respective spectra were adjusted by subtracting the buffer blank contribution and the buffer containing a specific amount of VPg, when applicable. All the spectral data were processed and averaged using the online server Dichroweb (http://dichroweb.cryst.bbk.ac.uk/html/links.shtml) [50]. CDNN software was utilized to calculate the change in secondary structure content of the phosphorylated eIFiso4Ep with addition of eIF4B (eIFiso4Fp∙eIF4B), and eIF4B and PABP (eIFiso4Fp∙eIF4B∙PABP) in the absence and presence of VPg. Secondary structure contents of the eIFiso4Fp were also estimated by the predictive methods, as described previously [51, 52]. The α-helical content of the eIFiso4Fp was estimated by Dichroweb agrees well with the value estimated from the mean residue ellipticity (MRE) at 222 nm, according to the equation:

$$\alpha ‐helix(\%)=(–MRE_{222}-2340)/30300\;X\;100$$ (Eq 3)

## Results

### PABP stimulates VPg-dependent TEV mRNA protein synthesis

**Fig 1:**
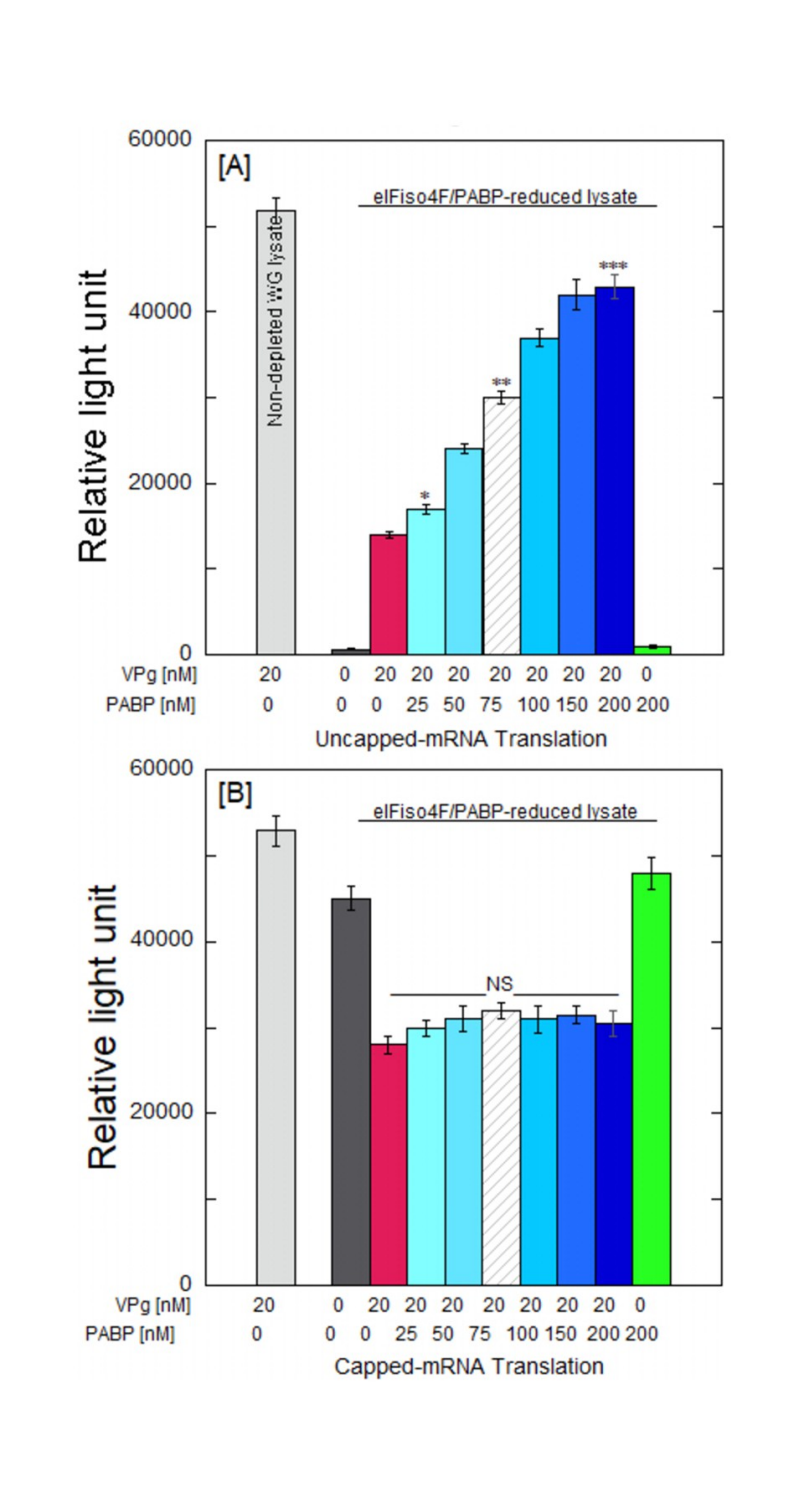
eIFiso4F/PABP-depleted WGE was programmed with VPg and uncapped or capped TEV-luc-A_50_mRNA. eIFiso4F mediates VPg dependent TEV translation. PABP stimulated VPg dependent TEV function. PABP-depleted WGE was programmed with uncapped [A] or capped [B] TEV mRNA and supplemented with the indicated amount of PABP. VPg concentration was 20 nM. Every reaction was carried out three times, and the average of the three outcomes was obtained. Standard deviation is shown as error bars. Statistical analysis was performed with a two-tailed Student’s t-test. *P<0.05, **P<0.02, ***P<0.001 as compared to control (no PABP, VPg alone). NS, no significance.

We have shown previously [28] that phosphorylated eIFiso4E strongly interacts with VPg of turnip mosaic virus. Phosphorylation increased the equilibrium binding affinity of eIFiso4E to VPg and activated VPg function in *in vitro* translation assays, which could rescue TEV-mediated translation in eIFiso4F-depleted WGE [28]. In this study, the translation stimulation efficiencies of phosphorylated eIFiso4Fp, eIF4B, and PABP were determined. A translation reporter construct utilizing the TEV 5’-untranslated region (UTR) to flank the firefly luciferase reporter gene, as previously described [16], was employed. To investigate whether VPg-dependent translation would be increased by the TEV_1-43_ 5’-UTR in eIFiso4F/eIF4F- reduced lysate, *luc* mRNA with the TEV 5’-UTR(TEV-*luc*-A_50_) was translated. The constructs were transcribed to generate both capped or uncapped mRNA, and the extent of translation of the reporter mRNA was evaluated by quantifying luciferase activity. To assess the effects of eIF4F/eIFiso4F in translation, it was crucial to create eIF4F/eIFiso4F/4B/PABP reduced WGE. The abundance of the initiation factors eIF4F (comprised of eIF4E and eIF4G subunits) and eIFiso4F (comprised of eIFiso4E and eIFiso4G subunits) bind m7GTP, the level of both factors in WGE was decreased by their association with m^7^GTP-sepharose. Western blot (S1 Fig) confirmed that the levels of eIF4E, eIFiso4E, eIF4G, eIFiso4G, eIF4B, and PABP were reduced by about 90% following resolution of the extract by running 10% SDS-PAGE as demonstrated previously [10, 26]. Fig 1 shows the effect of PABP on the stimulation of VPg-dependent translation in the PABP/eIF4B/eIF4F/eIFiso4F-depleted WGE, along with the addition of escalating concentrations of purified PABP. The addition of VPg enhanced uncapped TEV mRNA translation ~20-fold in eIF4F/eIFiso4F/eIF4B/PABP-depleted WGE. The addition of 200 nM PABP further enhanced translation (3.0-fold) for the uncapped mRNA in the presence of 20 nM VPg as compared to the absence of PABP (Fig 1A). However, VPg significantly inhibited translation for capped mRNA in eIF4F/eIFiso4F/eIF4B/PABP-depleted WGE. Further addition of PABP did not affect the translation of capped mRNA construct in the presence of VPg (Fig 1B). These data illustrate that PABP promotes uncapped TEV mRNA translation as compared to capped mRNA with the addition of VPg in eIF4F/PABP depleted WGE. VPg provides viral RNA with a substantial competitive advantage by promoting the translation of uncapped mRNA and suppressing the translation of capped mRNA.

**Fig 2:**
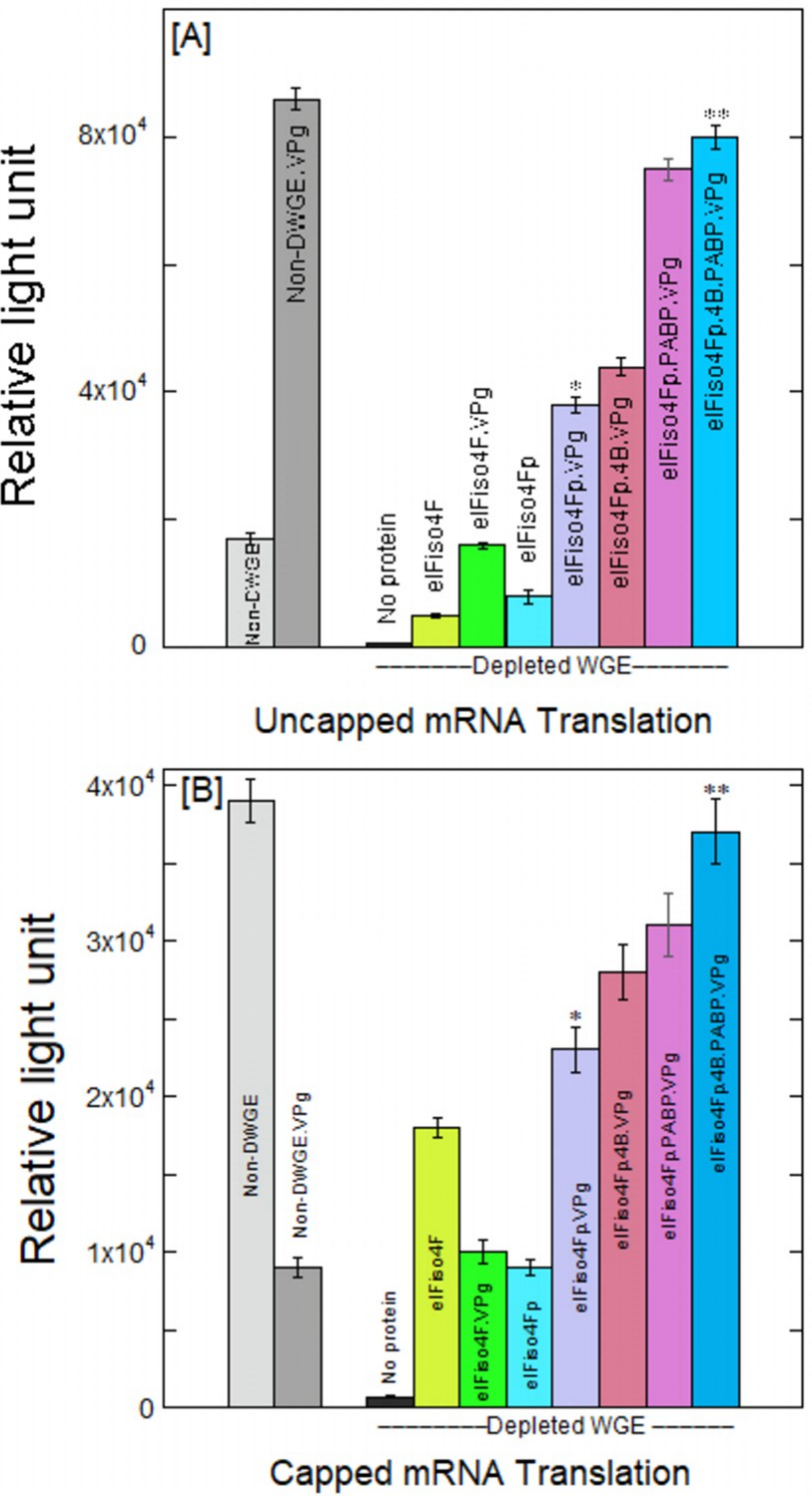
eIF4F/eIFiso4F/PABP-depleted WGE was programmed with uncapped [A] or capped [B] TEV-luc-A_50_mRNA. Depleted WGE was supplemented with 50 nM eIFiso4Fp, 100 nM eIF4B, 100 nM PABP, and 20 nM VPg, respectively. Three duplicates of each reaction were run, and the average of the three was obtained. Standard deviation is represented by error bars. Statistical analysis was performed with a 2-tailed Student’s t-test. *P<0.02, **P<0.01 as compared to control (eIFiso4Fp alone).

**Fig 3:**
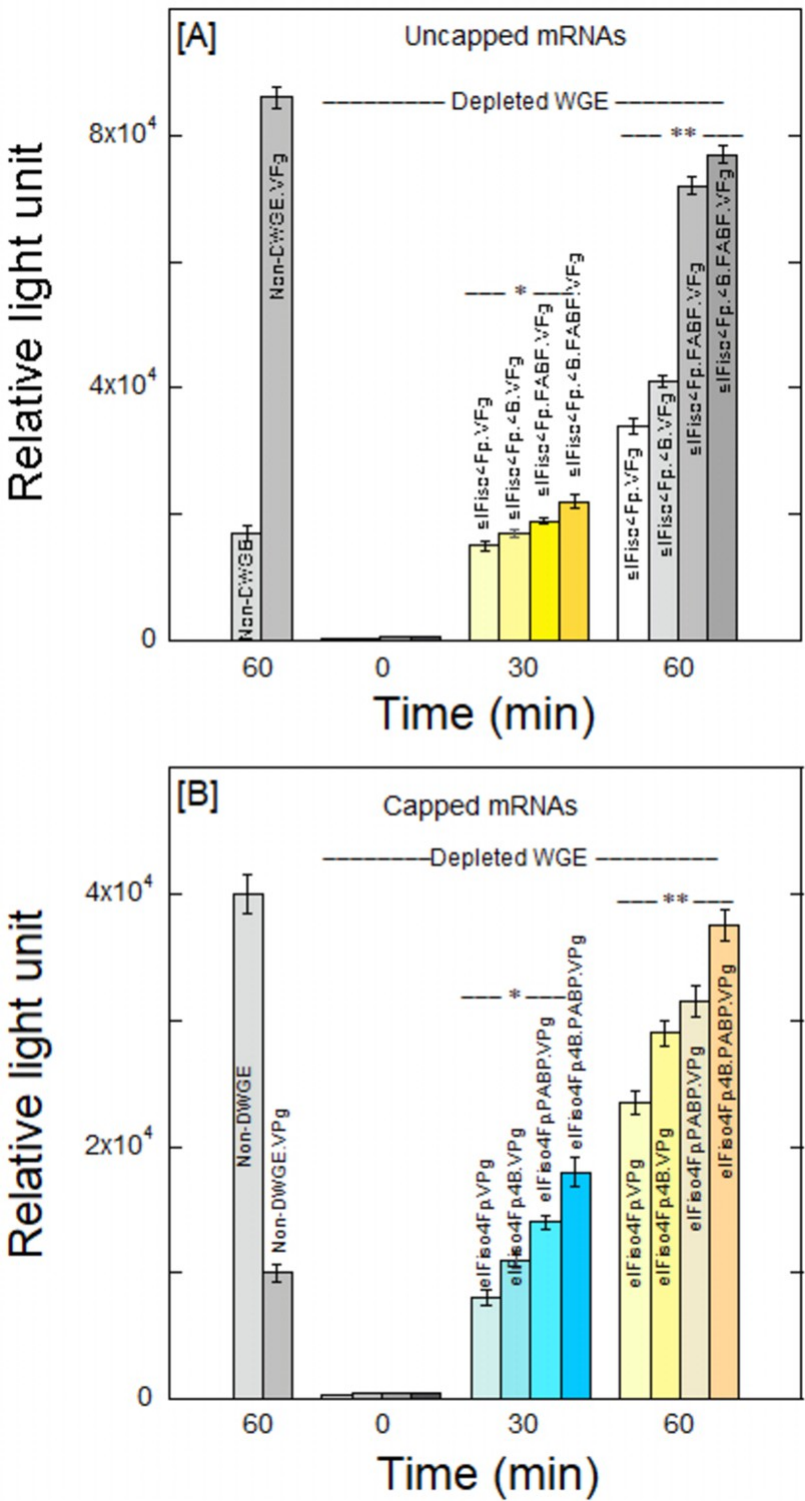
Function of PABP in the translation of uncapped and capped TEV mRNA in eIFiso4F depleted WGE. eIFiso4F/4B/PABP depleted WGE was mixed with 1.0 μg uncapped TEV RNA (A) and 1.0 μg capped TEV RNA (B), in the presence of 50 nM eIFiso4F, 100 nM (each) eIF4B, PABP, and 20 nM VPg, respectively, in a luciferase assay buffer. The light emission was measured after the addition of 0.5 mM luciferin. We carried out each reaction three times and averaged the results. The standard deviation is shown by error bars. *P<0.02, **P<0.01 from control (zero time), as determined by a 2-tailed Student t-test.

Further, to investigate whether VPg-dependent translation would be increased by the phosphorylation in the eIF4F/eIFiso4F-depleted WGE, *luc* mRNA with TEV 5’-leader was translated. The constructs were translated as capped or uncapped mRNAs, and the level of translation for each reporter mRNA was assessed by measuring luciferase activity. Fig 2A and 2B, shows the histograms for the translational efficiency of capped and uncapped mRNA with non-depleted and depleted eIF4F/eIFiso4F/4B/PABP WGE in the presence and exclusion of VPg. Depleted WGE shows about 122- and 55-fold decrease in translation as compared to non-depleted WGE for uncapped (Fig 2A) and capped TEV mRNA (Fig 2B). When non-depleted WGE was used to program mRNA, translation from uncapped mRNA exhibited a 2-fold increase compared to that from capped mRNA (Fig 2A and 2B). The addition of VPg to the non-depleted wheat lysate containing uncapped mRNA constructs enhances translation about 5-fold. In contrast, the addition of VPg results in a 4-fold inhibition of translation for capped mRNA in non-depleted WGE. Nevertheless, in DWGE, translation was enhanced by approximately 26 times when unphosphorylated eIFiso4F was added to capped mRNA. Moreover, VPg supplementation reduces DWGE inclusion with eIFiso4F by almost a factor of two in cap-dependent translation (Fig 2B). The presence of eIFiso4F showed about an 8-fold increase in the translation of uncapped mRNA in depleted WGE. Supplementation of phosphorylated eIFiso4F has a minimal impact on the translation of uncapped mRNA in contrast to unphosphorylated eIFiso4F. The addition of VPg enhances translation 5-fold for uncapped mRNA in depleted lysate with the supplementation of phosphorylated eIFiso4Fp (eIFiso4Ep and eIFiso4Gp) (Fig 2A). Furthermore, the addition of eIF4B in depleted WGE containing eIFiso4Fp has little effect on the translation of VPg-dependent uncapped mRNA. The addition of PABP in depleted WGE containing eIFiso4Fp and VPg increases protein synthesis 2-fold. The inclusion of VPg to phosphorylated eIFiso4F along with eIF4B and PABP in the depleted WGE was able to restore the translation of uncapped mRNA. In contrast, the introduction of phosphorylated eIFiso4F to capped mRNA in depleted WGE reduces translation by 2-fold compared to unphosphorylated eIFiso4F (Fig 2B). However, the incorporation of VPg into capped mRNA in depleted WGE supplemented with eIFiso4Fp enhances translation about 2.5-fold. VPg was able to overcome the inhibition of cap-dependent translation by being added to phosphorylated eIFiso4F together with eIF4B and PABP in depleted WGE, hence enhancing VPg-dependent translation (Fig 2B). These results suggest that VPg supports the translation of both uncapped and capped TEV mRNA under phosphorylation states. In a previous study by Cotton *et al*. [53], it was observed that VPgPro suppresses the translation of capped mRNA in WGE and rabbit reticulocyte lysate. Our findings align with the observed inhibition of capped mRNA translation and additionally reveal that VPg promotes the translation of uncapped TEV mRNA and overcomes the suppression of capped mRNA translation under phosphorylation states in WGE. Fig 3A and 3B showed the luciferase activity at different times for VPg-dependent uncapped and capped mRNA translation in depleted WGE supplemented with eIFiso4Fp, eIF4B, and PABP. After 60 min reaction, the uncapped (Fig 3A) and capped (Fig 3B) mRNA translation was rescued by supplementation of eIFiso4F, eIF4B, and PABP in VPg-dependent translation was close to saturation. These data suggest that phosphorylation of initiation factor eIFiso4Fp can bypass the translational impediment of capped TEV mRNA and promotes efficient VPg-dependent viral translation.

## PABP effect on the binding of phosphorylated eIFiso4F and VPg

**Fig 4:**
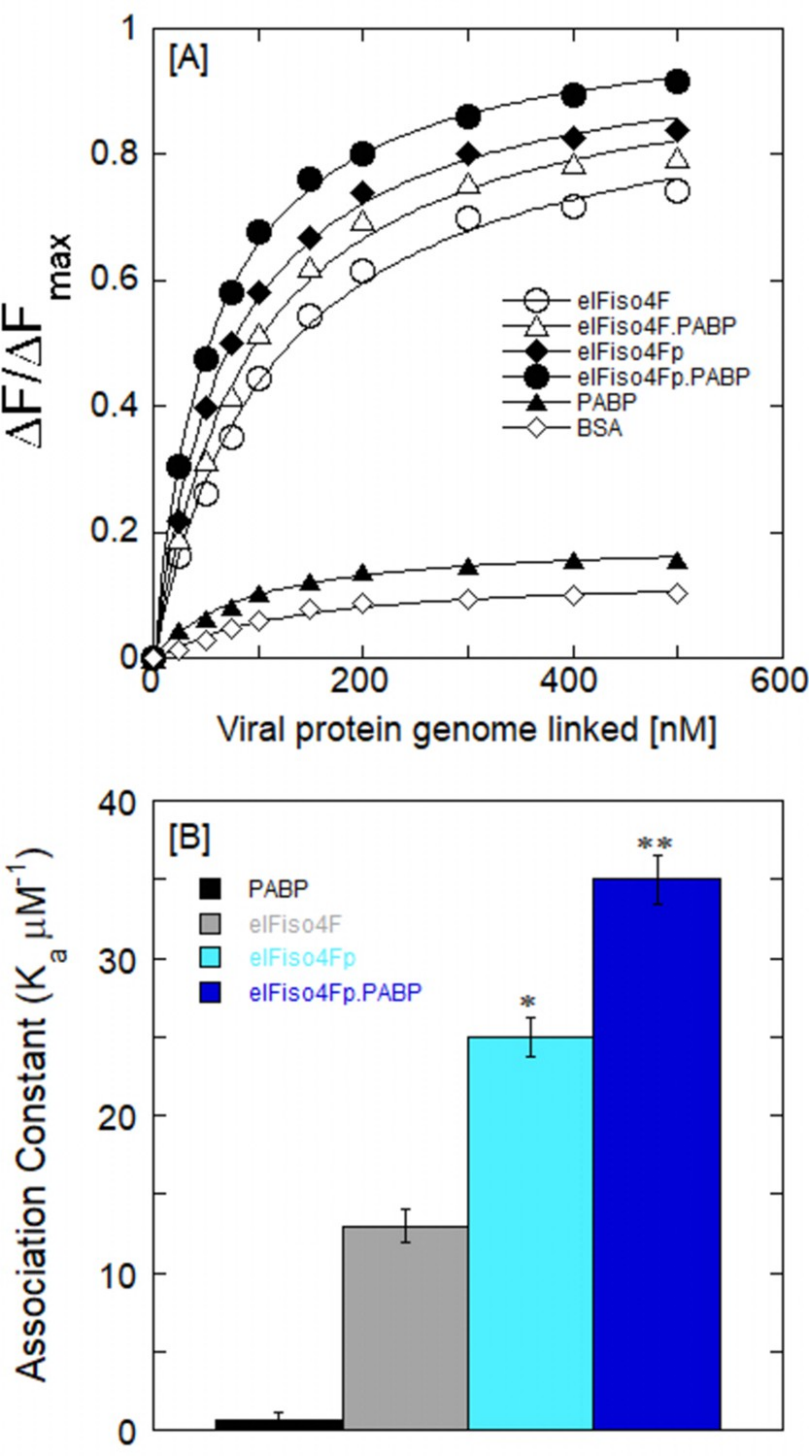
**Poly(A)-binding protein enhances the equilibrium binding of phosphorylated eIFiso4F with VPg.** (A) The ΔF/ΔF_max_ values of eIFiso4F•VPg (—О—), eIFiso4F•PABP•VPg (−Δ−), eIFiso4Fp•VPg (—♦—), eIFiso4Fp•PABP•VPg (—●—), PABP•VPg (—▲—) and BSA•VPg (−◊−) are shown. Fluorescence intensity for the binding of 100 nM eIFs or BSA with varying amount of VPg were measured. Initiation factor eIFiso4Fp:PABP (1:10) complex was prepared by incubation of 0.1 μM eIFiso4Fp and 1 μM PABP for 15 min at 25°C. The excitation and emission wavelengths were 280 nm and 332 nm, respectively. The dissociation constant (K_d_) was obtained from the fit curve as described under “Materials and Methods”. (B) Histogram representation of binding affinity of eIFiso4F, eIFiso4Fp, eIFiso4Fp•PABP, and PABP with VPg. Values are means of three replicates for each experiment. Error bars indicate SD. Statistical analysis was performed with a two-tailed Student’s t-test. *P<0.02, **P<0.01 significantly different from control (eIFiso4F or PABP alone).

Previously, we demonstrated the interaction of eIFiso4F with VPg [19, 26, 27]. In this study, we investigated the impact of PABP on the phosphorylated eIFiso4Fp interaction with VPg of turnip mosaic virus. The comparison of VPg binding affinity for the phosphorylated and unphosphorylated eIFiso4F by means of direct fluorescence titration experiments could elucidate the function of protein modification in the translation initiation mechanism. The binding of phosphorylated and unphosphorylated forms of eIFiso4F with VPg was studied by fluorescence quenching experiments as shown in Fig 4A. To examine the binding affinity of VPg with phosphorylated and unphosphorylated eIFiso4F, 100 nM eIFiso4F or eIFiso4Fp were titrated with varying concentrations of VPg. Phosphorylation of eIFiso4Fp enhances the binding affinity for the VPg of turnip mosaic virus. The binding of eIFiso4F to VPg was affected by phosphorylation as demonstrated with the fluorescence intensity plots of Fig 4A and Table 1 (*K*_d_ = 41.0 ± 3.1 nM for eIFiso4Fp•VPg, *K*_d_ = 81.3 ± 1.9 nM for eIFiso4F•VPg). Fig 4B shows the binding affinity plots for the various initiation factors with VPg. The binding of phosphorylated eIFiso4Fp is 2-fold higher, in relation to the un-phosphorylated eIFiso4F with VPg at 25°C. Binding data indicate that phosphorylation substantially increased the binding affinity of the eIFiso4F•VPg complex in comparison to its un-phosphorylated form.

**Table 1:** PABP enhances binding affinity between viral protein-genome linked (VPg) with phosphorylated eIFiso4F and eIFiso4Fp∙eIF4B.

Complex	*K*_d_ [nM]				
	5°C	10°C	15°C	20°C	25°C
eIFiso4F•VPg*	30.5 ± 0.4	39.2 ± 0.3	47.9 ± 0.4	61 ± 0.5	81.3 ± 1.9
eIFiso4Fp•VPg	17.1 ± 2.3	21.0 ± 3.3	26.2± 3.9	31.0 ± 1.4	41.0 ± 3.1
eIFiso4Fp∙4B•VPg	15 ± 2.5	19.1 ± 1.9	24.0 ± 1.5	29.3 ± 2.8	34.1 ± 1.6
eIFiso4Fp∙PABP•VPg	10.3 ± 0.5	14.0 ± 1.2	19.1 ± 0.9	26.5 ± 2.9	32 ± 3.3
eIFiso4Fp∙4B∙PABP•VPg	8.6 ± 0.2	12.0 ± 0.8	16.1 ± 1.5	19.5 ± 0.9	24.0 ± 1.7

*Values were obtained from reference [19].

**Fig 5:**
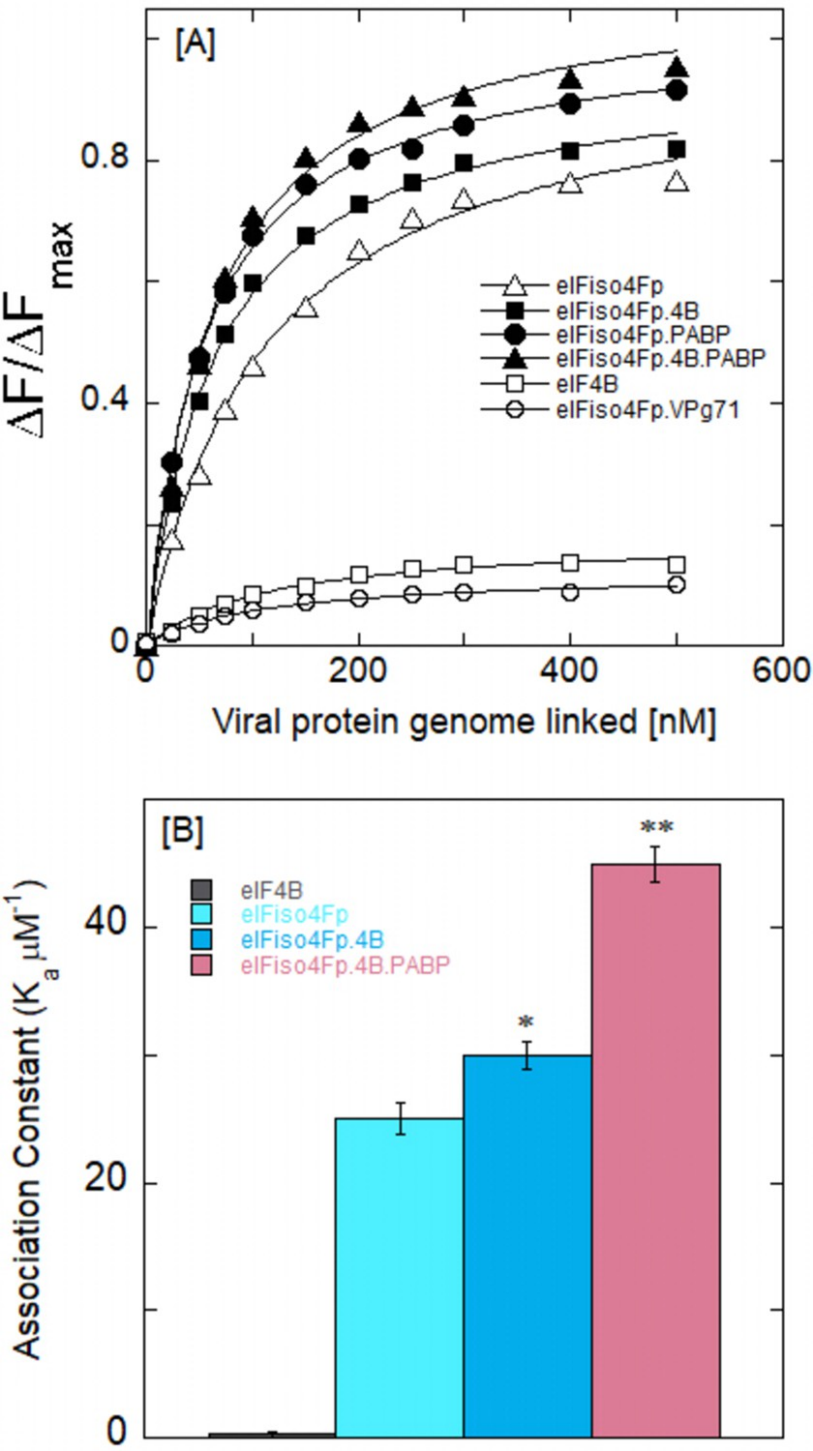
PABP affects the binding of eIFiso4Fp•4B with VPg. (A) The normalized fluorescence intensity for the binding of eIFiso4Fp•VPg (—Δ—), eIFiso4Fp•4B•VPg (—■—), eIFiso4Fp•PABP•VPg (−•−), eIFiso4Fp•4B•PABP•VPg (—▲—), eIF4B•VPg (—□—) and eIFiso4Fp•VPg_71_ (−Ο−) are shown. Initiation factors eIFiso4F:eIF4B (1:10), eIFiso4Fp:4B: PABP (1:10:30) complex were prepared by incubation of 0.1 μM eIFiso4Fp and 1 μM eIF4B, and 3 μM PABP for 15 min at 25°C. Experimental conditions were as described in the legend of Fig 4. (B) Histogram representation of binding affinity of eIFiso4Fp, eIFiso4Fp•eIF4B, eIFiso4Fp•eIF4B•PABP, and eIF4B with VPg. The data are the averages of three independent times and analysis with the error as the standard deviation. *P<0.05, **P<0.01 from control (eIFiso4Fp or eIF4B alone), as determined by a 2-tailed Student’s t-test.

Since eIFiso4F interacts with other initiation factors *in vivo*, it was important to examine whether the phosphorylation of eIFiso4F affects the binding to VPg in the presence of PABP and eIF4B. Therefore, it was of interest to assess the interaction between phosphorylated eIFiso4Fp and VPg, and to investigate how this interaction is modulated by the presence of PABP and eIF4B within the initiation factor complex. Figs 4A and 5A demonstrate a representative binding plot ΔF/ΔF_max_ *versus* VPg concentration for determination of the equilibrium binding constant. PABP significantly enhanced the binding affinity of phosphorylated eIFiso4F with potyvirus VPg (eIFiso4Fp∙PABP•VPg, *K*_d_ = 32 ± 3.3 nM) (Figs 4A and 5B). However, the inclusion of both eIF4B and PABP with eIFiso4Fp enhanced binding affinity about 2-fold (eIFiso4Fp∙eIF4B∙PABP•VPg, *K*_d_ = 24 ± 1.7 nM) (Fig 5A and 5B, Table 1). Fig 5B shows the corresponding binding affinity plots for the interaction of eIFiso4Fp with VPg in the presence of eIF4B and PABP. The addition of eIF4B has minimal impact on the binding between eIFiso4Fp and VPg (Fig 5B). Further, very weak interaction was detected between PABP and eIF4B with potyvirus VPg (Figs 4 and 5). Because the binding of eIF4B and PABP alone was very weak, an abundant supply of both proteins was added to eIFiso4F, ensuring that at least 90% of the eIFiso4F existed in complex form (eIFiso4Fp∙eIF4B, eIFiso4Fp∙PABP, and eIFiso4Fp∙eIF4B∙PABP), taking into account the predicted concentrations based on the *K*_d_ values [14, 54] for the protein-protein interactions. This data indicates that PABP can increase VPg activity in a way that is comparable to how it affects a 5’-cap. VPg71 was utilized in previous research to determine whether this was a unique impact of VPg and whether it was connected to the interaction with eIFiso4F. VPg71, a truncated variant of VPg (N-terminal 1–70 amino acids), is incapable of interacting with either eIFiso4E or eIF4E [54]. We investigated VPg71 binding to eIFiso4Fp. As anticipated, there was no change in fluorescence for VPg71 with eIFiso4Fp (Fig 5). It has been previously demonstrated that VPg71 does not bind to eIFiso4F [26]. In addition, BSA was tested for non-specific binding under the same circumstances as a control. When VPg was added, the fluorescence intensity for BSA did not alter noticeably (Fig 4). Although eIF4B and PABP do not interact directly with the VPg, it stimulates eIFiso4Fp-dependent VPg-mediated translation.

### PABP-induced conformational changes in eIFiso4Fp and increases their binding to the VPg

**Fig 6:**
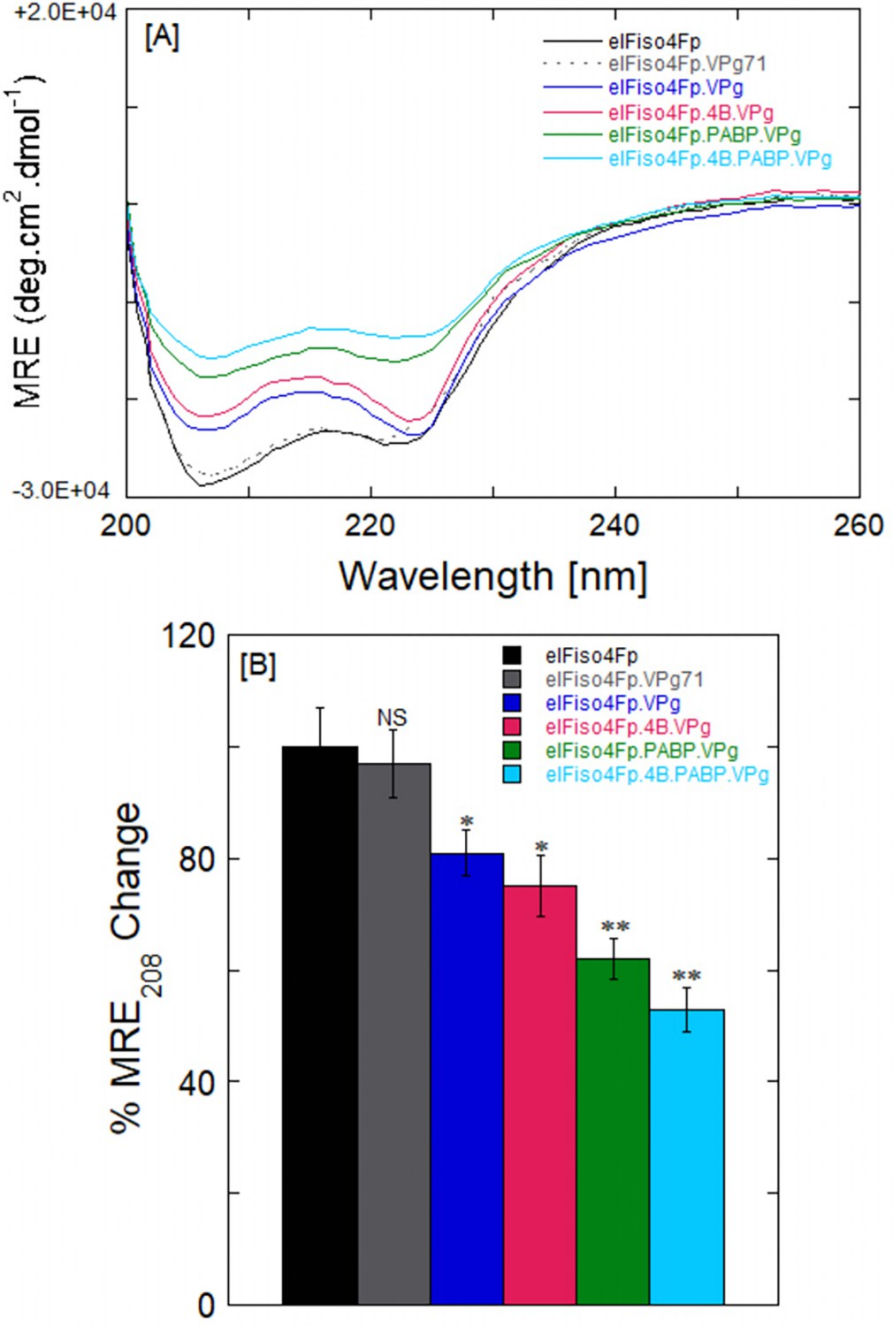
CD spectra of eIFiso4Fp with VPg. The far-UV CD spectra of eIFiso4Fp with VPg were obtained in the presence of eIF4B and PABP. VPg induces statistically significant helical changes in eIFs protein complex. (A) CD spectra are shown for eIFiso4Fp (0.1 μM) with VPg (1 μM) in the presence of eIF4B (1 μM) and PABP (10 μM). Control experiment was run for eIFiso4Fp with mutant VPg71 to determine the specificity of the binding. Each data point corresponds to the mean ± SD, n = 3. (B) Bar graph comparing the spectral minimal MRE_208nm_ value is shown. Mean and SD are shown. The data are the results of triplicate experiments. Statistical analysis was performed with a two-tailed Student’s t-test. *P<0.05, **P<0.02 as compared to control (eIFiso4Fp alone). NS, no significance.

To examine the possible PABP-induced conformational changes in eIFiso4Fp and formed a protein-protein complex with various initiation factors (eIF4B, PABP) and increase their binding to the VPg, CD measurements in the far-UV region were used to analyze the changes in the secondary structure of the protein-protein complex. In this investigation, we examined the CD spectrum of eIFiso4Fp alone, and subsequently explored the alterations in conformation observed in eIFiso4Fp∙4B, eIFiso4Fp∙PABP, and eIFiso4Fp∙4B∙PABP when they formed a protein complex with VPg. The far-UV CD spectra of eIFiso4Fp in complex with VPg, accompanied by the presence of eIF4B and PABP, are illustrated in Fig 6. Phosphorylated eIFiso4Fp protein exhibits CD spectra with prominent minima at 208 and 222 nm, indicating the presence of an alpha-helical conformation. With the addition of VPg to the initiation factors complex leads to an increase in ellipticity at 208 and 222 nm, indicating a decrease in alpha-helical content and a corresponding increase in beta-sheet formation. The presence of a significant fraction of the alpha-helix is indicated by the presence of minima at 208 and 222 nm in the CD spectra [23, 51]. CD spectra of the eIFiso4Fp∙4B∙PABP complex with VPg produce a nearly flattened profile at 208 and 222 nm, indicating that the structural elements within the initiation factors complex have assumed a conformation different from the dominant alpha-helix. The spectral flattening observed between the closely positioned minima at 208 and 222 nm is a distinctive characteristic of the alpha/beta structural class, which encompasses intermixed alpha-helical and beta-sheet structural domains [55]. Fig 6B shows the bar plots for the change in ellipticity value at 208 nm for the binding of eIFiso4Fp with VPg in the presence of eIF4B and PABP. The addition of VPg to the eIFiso4Fp in the presence of eIF4B and PABP led to a significant modification in the CD readings at 208 and 222 nm wavelengths, signifying a secondary structural transformation in protein-protein complex and implied the formation of a complex between eIFiso4Fp∙4B∙PABP and VPg. The addition of VPg, eIF4B, and PABP induces changes in the secondary structure content, causing the initially less ordered alpha-helical structure of the initiation factors-VPg proteins complex (eIFiso4Fp∙4B∙PABP∙VPg) to fold into an active beta-sheet configuration. The estimation of secondary structural contents was performed using the previously described method [56]. The binding of VPg to eIFiso4Fp resulted in a reduction of the alpha-helix content to approximately 45% while increasing the beta-sheet content to approximately 35%. The addition of VPg led to a decrease in the alpha-helicity values of eIFiso4Fp. The observed changes in secondary structure indicate that eIFiso4Fp experiences certain conformational modifications upon the addition of VPg in the presence of eIF4B and PABP. PABP changes the conformation of eIFiso4Fp and increases their binding affinity to the VPg. It has been shown that the addition of other initiation factors (eIF4B, eIF4A) causes a conformational change in eIF4F/eIFiso4F [57]. This large structural alteration suggests eIFiso4Fp∙4B∙PABP complex strongly interacts with VPg potentially mediated by the beta-sheet motif and indicates a potential regulatory role for this conformational transition. PABP and eIF4B effect of increasing the binding between eIFiso4Fp and VPg. PABP and eIF4B may act as a molecular chaperone for its partner eIFiso4F, inducing an overall secondary structure change of eIFiso4F along with eIF4B, PABP and VPg in the reaction mixture with less helix content and possibly exposing more eIFiso4Fp regions to VPg. VPg’s specificity was assessed using a truncated variant of wild type VPg71 [25]. According to prior research, VPg71 does not bind to eIFiso4E or eIF4E [25, 26]. Run as control trials, eIFiso4Fp with VPg71 showed no significant difference in conformational change or binding to eIFiso4Fp (Fig 6).

### The binding of eIFiso4Fp with VPg is both enthalpically and entropically favorable

**Fig 7:**
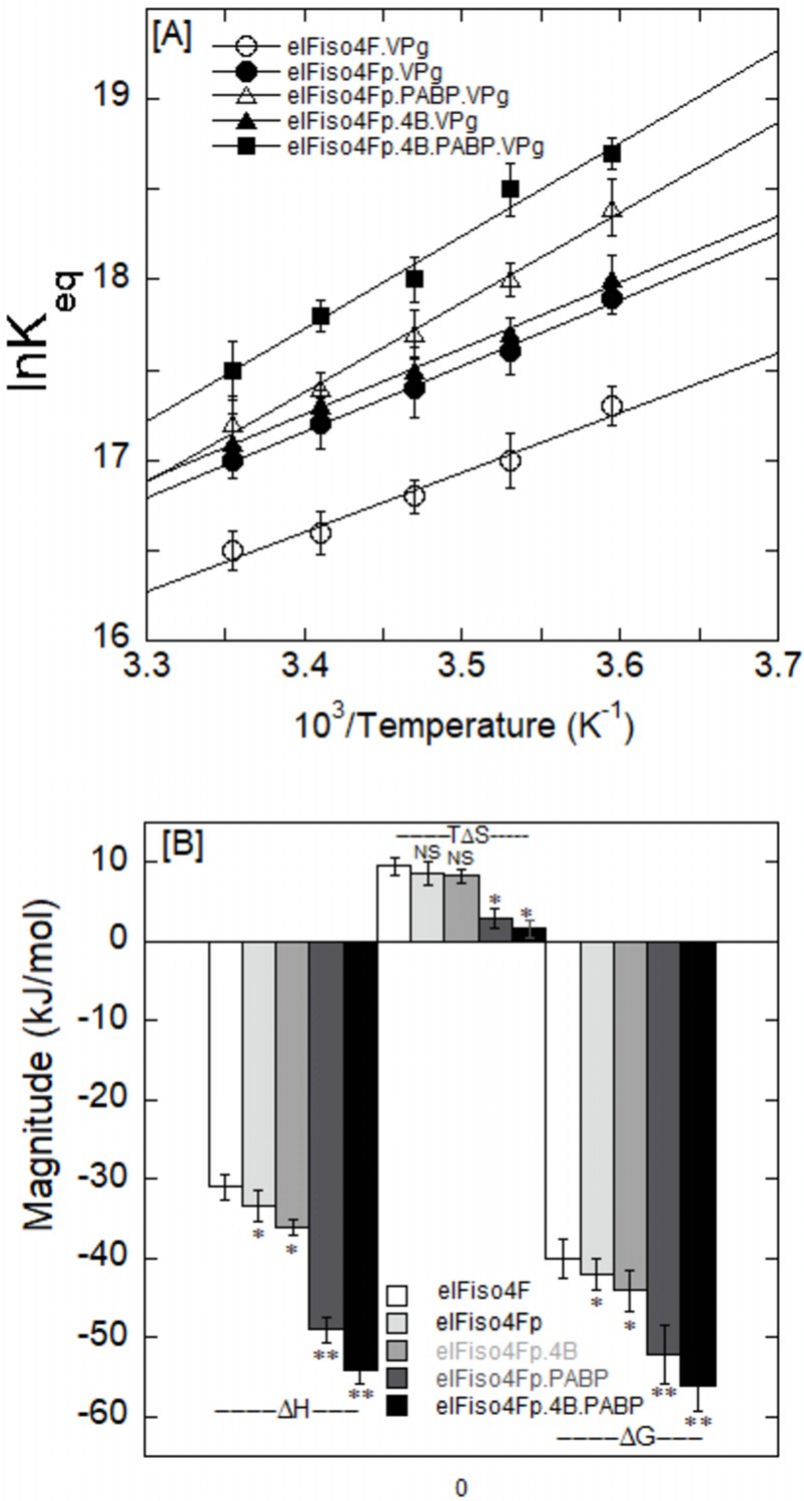
Thermodynamics of eIFiso4Fp interaction with VPg with addition of eIF4B and PABP. (A) The data are indicated as, eIFiso4F•VPg (—О—), eIFiso4Fp•VPg (—●—), eIFiso4F•PABP•VPg (—Δ—), eIFiso4Fp•4B•VPg (—▲—), and eIFiso4Fp•4B•PABP•VPg (—■—), respectively. Enthalpy (ΔH) and entropy (ΔS) were calculated from the slope and intercept of the temperature dependent Van’t Hoff plot. Equilibrium values were averaged from three independent individual experiments. (B) Thermodynamic parameters of the eIFs binding to VPg. The bar diagram shows the change in free energy (ΔG), enthalpy (ΔH) and in the contribution of entropy to the free energy (-TΔS) of the binding of eIFs to the VPg. Each reaction was performed in triplicate, and the results were averaged. Error bars represent standard deviation. *P<0.05, **P<0.01 from control (eIFiso4F), as determined by a two-tailed Student t-test. NS, no significance.

The equilibrium dissociation constants at different temperatures were measured (Table 1), and thermodynamic studies for the phosphorylated eIFiso4Fp with VPg in the presence of PABP and eIF4B were performed. The enthalpy (ΔH) and entropy (ΔS) were obtained by analyzing Van’t Hoff plots of ln*K*_eq_ *versus* the reciprocal of temperature (1/T) as presented in Fig 7A. The ΔH and ΔS values were determined from the slope and intercept of the data, respectively, and are presented in Table 2. The van’t Hoff analyses demonstrated that the VPg binding to eIFiso4Fp was both enthalpically and entropy-favorable with a large negative ΔH (–33.4 ± 2.3 kJ mol^-1^) and positive ΔS (29.0 ± 1.4 J mol^-1^ K^-1^) (Table 2 and Fig 7A). Free energy (ΔG), values were calculated at 25°C using Eq (2) and Table 2 values. Fig 7B shows the thermodynamic parameters of the eIFiso4Fp binding to VPg in the presence of eIF4B and PABP. The eIFiso4Fp interaction to VPg is both enthalpically (79.5%) and entropically (20.6%) favorable with approximately 60% more enthalpic contribution to ΔG at 25°C. The addition of PABP and eIF4B increased the enthalpy contribution to 97% and reduced the entropic contribution to 3.9% in the binding of eIFiso4Fp to VPg. eIFiso4Fp∙4B∙PABP has a greater enthalpic contribution to ΔG than the eIFiso4F interaction with VPg [19]. The increased enthalpic value indicates the presence of extra hydrogen bonds between the eIFiso4Fp and VPg in the presence of PABP and eIF4B. The magnitude and sign of the thermodynamic parameters contributed to the involvement of the binding forces between protein-protein and protein-RNA [44, 49, 58]. The addition of PABP and eIF4B increased the free energy, ΔG, for eIFiso4Fp binding to VPg (ΔG = – 55.3 ± 1.3 kJ/mol for eIFiso4Fp∙4B∙PABP•VPg, ΔG = – 42.0 ± 1.5 kJ/mol for eIFiso4Fp•VPg). The presence of eIF4B has minimal impact on the enthalpic and entropic contribution to the interaction between eIFiso4Fp and VPg. PABP enhances the enthalpic contribution by about 94.5% and 97% and lowers the entropic contribution to about 8% and 4% for the interaction of eIFiso4Fp and eIFiso4Fp∙4B with VPg. These results imply that the presence of poly(A)-binding protein triggers a conformational change within the initiation factor complex, leading to strengthened hydrogen bonding and weakened hydrophobic interactions, which could heighten interaction specificity.

**Table 2:** Thermodynamic analysis for the binding of viral protein-genome linked, VPg, with phosphorylated eIFiso4F complex with eIF4B and PABP.

Complex	Δ*H* kJ/mole	Δ*S* J/mole/K	Δ*G* kJ/mole	TΔS/ΔG %
eIFiso4F•VPg*	–31.0 ± 0.2	32.3 ± 0.4	–40.4 ± 0.5	24.0
eIFiso4Fp•VPg	–33.4 ± 2.3	29.0 ± 1.4	–42.0 ± 1.5	20.6
eIFiso4Fp∙4B•VPg	–35.4 ± 2.0	28.0 ± 2.7	–43.7 ± 0.9	19.0
eIFiso4Fp∙PABP•VPg	–48.9 ± 2.5	13.4 ± 0.7	–51.8 ± 1.7	7.7
eIFiso4Fp∙4B·PABP•VPg	–53.7 ± 3.2	7.3 ± 0.2	–55.3 ± 1.3	3.9

*Values were obtained from reference [19].

## Discussion

In this study, we have shown that the equilibrium binding of phosphorylated eIFiso4F to potyvirus VPg correlated well with the translation efficiencies. Viral infection has been shown to alter the phosphorylation status of plant initiation proteins [59]. The question of whether eIFiso4E or eIF4E are phosphorylated and the potential functional ramifications of this process remain unanswered. We looked at the impact of in vitro phosphorylation since it can be challenging to isolate significant amounts of these proteins from wheat sprouts for biophysical and biochemical investigations. There was no discernible change in the isoelectric focusing pattern after eIFiso4E was treated with casein kinase 2. On the other hand, the treatment’s functional effects were strikingly similar to those seen with mammalian eIF4E. Protein synthesis is increased when mammalian eIF4E is phosphorylated [60, 61]. Our findings suggest that the wheat germ translation machinery has a comparable effect. Phosphorylation of eIFiso4F substantially increases the *in vitro* translation of uncapped TEV mRNA and capped TEV mRNA translation in depleted WGE supplemented with initiation factor eIFiso4Fp and VPg together, in agreement with previous findings [28] indicating that potyvirus VPg affects the uncapped and capped protein synthesis. In non-depleted WGE, VPg promotes the translation of uncapped mRNA while suppressing the translation of capped mRNA. The addition of phosphorylated eIFiso4Fp to eIF4F/eIFiso4F depleted WGE resulted in increased protein synthesis of uncapped mRNA, while phosphorylation had an inhibitory effect on the protein synthesis of capped mRNA translation. The addition of VPg to the eIFiso4Fp enhanced the translation both uncapped and capped mRNA in depleted WGE, validating the role of eIFiso4F phosphorylation in VPg binding and promoting VPg-dependent viral translation. This data indicated that phosphorylation further stimulates VPg-dependent viral protein synthesis. The binding affinity for the mRNA cap is decreased by phosphorylation of eIFiso4F [31]. Reduced cap-binding affinity causes phosphorylation-induced reductions in cap-dependent translation. On the other hand, eIFiso4F phosphorylation raises the binding affinity for VPg. Changes in helicase activity are connected to affect mRNA selection based on the secondary structure in the non-coding region, and an increase in VPg binding affinity is linked to increased VPg-dependent mRNA translation. Alterations in cap-binding activity, phosphorylation, and VPg function as crucial regulators of mRNA translation and selection. Because a phosphate group is added to the eIFiso4F molecule after phosphorylation, the resulting negative charge increases [31, 62]. The negatively charged phosphate group residues of eIFiso4F and the phosphate chain of the cap experienced an increase in electrostatic repulsion because of the eIFiso4F molecule’s subsequent rise in negative charge. VPg can establish a stable translational complex, which reprogrammed the translational machinery and releases initiation factors from the cap translational complex. These interactions highlight the significance of protein phosphorylation and are essential for controlling the translation initiation phase. Even though TEV lacks a cap structure, the genomic mRNA of TEV is effectively translated using a 5’-leader sequence that recruits the translational machinery, enabling successful protein synthesis. Apart from the 5’-leader sequence, the presence of a VPg, which is covalently linked to the 5’-end of TEV or turnip mosaic virus genomic RNA (a potyvirus similar to TEV), has been demonstrated to interact with eIFiso4E. Besides facilitating cap-independent translation, the VPg promotes uncapped TEV mRNA translation [26]. We conducted additional investigations to explore the role of PABP in stimulating TEV translation, similar to its function in 5’-cap translation. This is attributed to PABP’s role in stabilizing the binding of eIFiso4F to the 5’-cap [13]. The enhancement of VPg-dependent TEV translation by PABP may be attributed to its ability to stabilize the functional interaction between eIFiso4F and VPg. Through supplementation of depleted WGE with initiation factors either individually or in combination, it was determined that phosphorylation of eIFiso4F plays a critical role in enabling VPg-dependent TEV function. The addition of eIF4B in conjunction with the initiation complex during translation had minimal impact on TEV function. TEV translational activity was significantly increased by the addition of poly(A)-binding protein. Given that PABP enhances the stability of eIF4F binding to the 5’-cap [13], it is anticipated that PABP will enhance the role of the cap in facilitating translation. The enhancement of VPg function by PABP could be attributed to the stabilization of the functional interaction between eIFiso4F and VPg. The decreased enhancement of capped mRNA translation by PABP may indicate competitive binding of VPg and capped mRNA to eIFiso4F. PABP may also compete with the other factors required for translation, leading to a reduced degree of stimulation for capped mRNA.

To further understand these interactions, fluorescence measurements were performed between phosphorylated eIFiso4F and potyvirus VPg in the presence of PABP and eIF4B. It has been shown that potyvirus VPg interacts with eIFiso4F, and this interaction is necessary for the virus infectivity through favoring viral protein synthesis [19, 26]. Phosphorylation increases the affinity of eIFiso4F for VPg. PABP further enhances the binding of eIFiso4Fp and eIFiso4Fp∙4B with VPg, resulting in more than a 3-fold increase compared to the binding of unphosphorylated eIFiso4F to VPg. These findings indicate that PABP plays a role in stabilizing the assembly of the initiation factor complex with VPg under phosphorylation states. The interaction between eIFiso4Fp and PABP as well as VPg could potentially facilitate the circularization of RNA during the translation process. Circularization is an essential requirement for the effective translation of cellular mRNAs [63], and it is also observed in the case of animal viral RNAs [64]. Formation of the VPg•PABP, as well as the potential VPg•eIFiso4Fp•PABP complex, could potentially bring the two ends of the viral RNA into proximity.

Thermodynamic results showed that phosphorylation significantly changes the enthalpic and entropic contributions for the binding of eIFiso4F with VPg in the presence of PABP and eIF4B. The addition of PABP resulted in an increased enthalpic contribution and a decreased entropic contribution for both eIFiso4Fp and eIFiso4Fp∙4B binding to VPg. The ΔH and ΔS values were –53.7 ± 3.2 kJ/mol and 7.3 ± 0.2 J/mol/K for the eIFiso4Fp∙4B∙PABP•VPg complex. This suggests hydrogen bonding may be involved in the direct binding of VPg or through conformational changes within the initiation factor complex. The large, favorable, enthalpic contributions to the eIFiso4Fp•4B•PABP•VPg interactions imply that hydrophobic residues in the complex structure become more exposed to the solvent. These interactions could arise from either base stacking interactions involving hydrophobic amino acids or because of conformational changes within the proteins themselves. Due to the lack of comprehensive structural information, it is difficult to ascertain the exact origin of these contributions to the stability of the protein-protein complex. Furthermore, the thermodynamic analysis revealed a higher enthalpic contribution (97% compared with 79%) to the free energy of the binding between eIFiso4Fp∙4B∙PABP, eIFiso4Fp, and VPg, indicating an increased formation of hydrogen bonds within the complex. This interaction shows a significantly higher level of favorability compared to the interaction of eIFiso4F with VPg or m^7^GTP. Whereas the binding of the cap is entropically driven due to significant conformational changes in the eIF4E subunit. However, VPg binds to the eIFiso4E subunit of eIFiso4F with comparable thermodynamic characteristics, indicating similar mechanisms of interaction. The free energy data for the contribution of hydrogen bonding between eIFiso4Fp and VPg further supports the enthalpy findings. PABP enhanced the binding affinity about 2-fold and the corresponding change in free energy (ΔG) is about 13.3 kJ/mol for eIFiso4Fp∙4B∙PABP•VPg, in agreement with previously reported findings for about three more hydrogen bonds formed between a protein and ligand [65]. This data indicates that the elevation in enthalpic and reduction in entropic contribution to free energy for eIFiso4Fp∙4B∙PABP binding to VPg signify strengthened in the number of hydrogen bonding and decreased hydrophobic interactions. With the addition of PABP, VPg forms a strong binding interaction with eIFiso4Fp, which is further stabilized by the formation of additional hydrogen bonds. Binding of PABP to the eIFiso4Fp complex resulted in a conformational change and facilitated circularization, a crucial process for efficient viral translation [21]. This conformational change can facilitate the assembly of either the ternary (eIFiso4Fp∙PABP•VPg) or quaternary (eIFiso4Fp∙4B∙PABP•VPg) complex. The formation of this complex could bring the two ends of the turnip mosaic viral RNA into proximity, promoting RNA circularization during translation. Phosphorylation has been shown to impact the conformational change of eIFiso4F [55]. Phosphorylation increases the binding between eIFiso4E and VPg through structural alterations similar to the cap-binding [66]. The spectrum of phosphorylated eIFiso4F is characterized by the presence of minima at 208 nm and 222 nm, representing the presence of alpha-helical content in the eIFiso4Fp protein. The addition of PABP to eIFiso4Fp and eIFiso4Fp∙4B with VPg complex induces conformational change via changes in alpha-helical content and beta-sheet content. Such a conformational change could potentially enhance the specificity of VPg binding to eIFiso4Fp in the presence of PABP. This conformational change could bring other initiation factors in close proximity to promote RNA circularization and VPg could impede endogenous translation [66] which leads to efficient viral replication. The binding of eIF4E from yeast induces a conformational change in eIF4G, leading to enhanced binding to the cap and playing a critical role in the growth and maintenance of polysomes *in vivo* [67]. PABP further enhanced the stability of eIFiso4Fp•VPg complex through conformational changes. These results reveal that PABP induces conformational changes for the interaction between eIFiso4Fp and VPg, exhibiting similarities to those observed during cap binding. This significant conformational transition suggests that eIFiso4Fp binds VPg through a beta-sheet motif, and this structural change may play a regulatory role. By combining quantitative data and conformational change analyses, valuable insights were gained into the nature of interactions and the role of phosphorylation in the assembly of the initiation complex, providing crucial information about its capacity to facilitate translation initiation. These findings provide additional evidence highlighting the significance of the interaction between PABP, eIF4B, phosphorylated eIFiso4Fp, and VPg in facilitating efficient translation.

## Supporting information

S1 Fig Depletion of eIFiso4F, eIF4F, eIF4B and PABP from wheat germ extract. WGE was incubated with m7-GTP Sepharose for 1h. Western analysis was performed to determine the level of eIF4E, eIF4G, eIFiso4E, eIFiso4G, eIF4B and PABP in depleted WGE relative to non-depleted WGE.
(TIF)

S1 File (ZIP)

## Abbreviations

eIFiso4Fp: phosphorylated eIFiso4F
eIF: eukaryotic initiation factor
VPg: viral protein linked to the genome
TEV: tobacco etch virus
DWGE: depleted wheat germ extract
