# Supplementary figures and images for: Poly(A)-binding protein promotes VPg-dependent translation of potyvirus through enhanced binding of phosphorylated eIFiso4F and eIFiso4F∙eIF4B
Source: PLoS One. 2024 May 2;19(5):e0300287. doi: 10.1371/journal.pone.0300287 (PMC11065315; doi:10.1371/journal.pone.0300287)

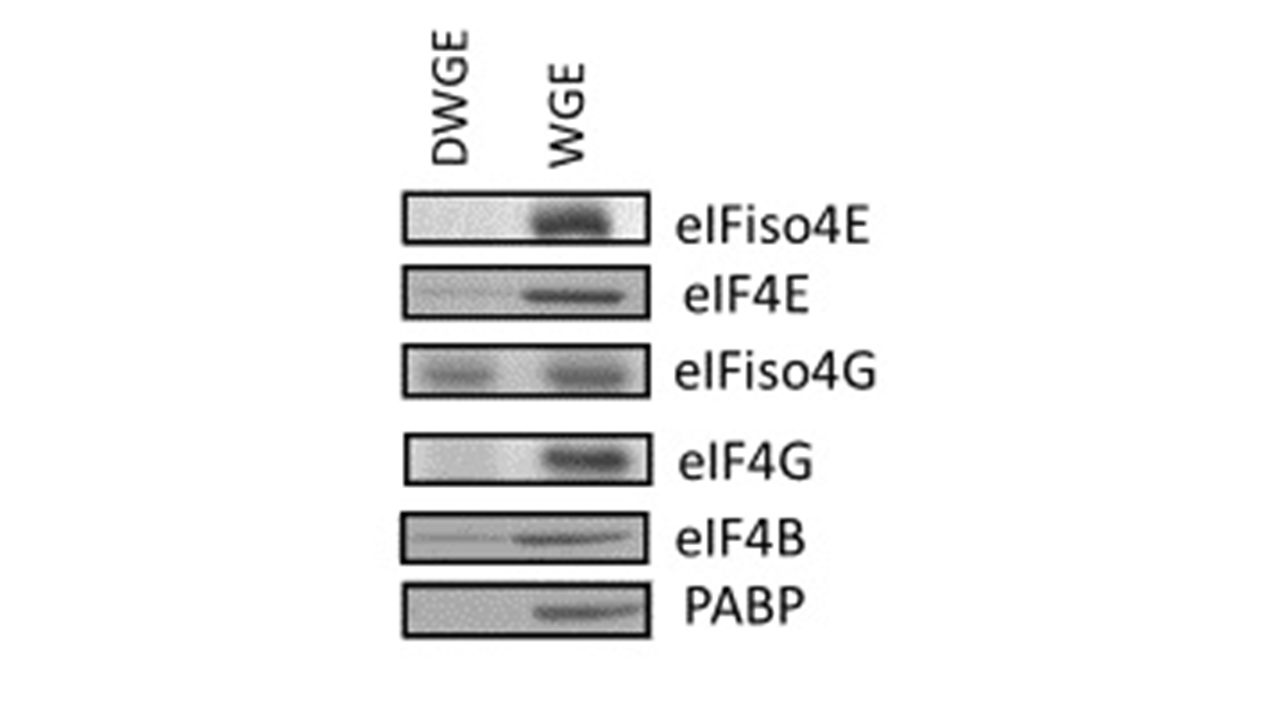

Supplement: S1 Fig — WGE was incubated with m7-GTP Sepharose for 1h. Western analysis was performed to determine the level of eIF4E, eIF4G, eIFiso4E, eIFiso4G, eIF4B and PABP in depleted WGE relative to non-depleted WGE. (TIF) [file pone.0300287.s001.tif]
