# Supplementary material for: Poly(A)-binding protein promotes VPg-dependent translation of potyvirus through enhanced binding of phosphorylated eIFiso4F and eIFiso4F∙eIF4B
Source: PLoS One. 2024 May 2;19(5):e0300287. doi: 10.1371/journal.pone.0300287 (PMC11065315; doi:10.1371/journal.pone.0300287)
Supplement: S1 File — (ZIP) [file pone.0300287.s002.zip › Data supporting information files/S1 Data Fig 1.pdf]

Non DWGE	iso4F-PABP red	20nMVPg a	20nMVPg,2	20VPg,50nl	20VPg,75nl	20VPg,100r	20VPg,150r	20VPg,200l
51896	600	14000	17000	23985	30000	37000	42000	42900

Error iso4F	Error 0VPg,	Error20VPg	Error 25pal	error 50pal	error75pab	error100pa	error150pa	error200pa
1000	1500	99	400	600	550	700	1100	1800

bp

1400 160
