# Supplementary material for: Poly(A)-binding protein promotes VPg-dependent translation of potyvirus through enhanced binding of phosphorylated eIFiso4F and eIFiso4F∙eIF4B
Source: PLoS One. 2024 May 2;19(5):e0300287. doi: 10.1371/journal.pone.0300287 (PMC11065315; doi:10.1371/journal.pone.0300287)
Supplement: S1 File — (ZIP) [file pone.0300287.s002.zip › Data supporting information files/S2 Data Fig 2.pdf]

Sample	RLU
nonDWGE	17000
nonDWGE.VPg	85896
no protein	600
eIFiso4F	5000
eIFiso4F+VPg	16000
eIFiso4Fp	8000
eIFiso4Fp+VPg	38000
eIFiso4Fp.4B.VPg	44000
eIFiso4Fp.PABP.VPg	75000
eIFiso4Fp.4B.PABP.VPg	80000
