# Supplementary material for: Poly(A)-binding protein promotes VPg-dependent translation of potyvirus through enhanced binding of phosphorylated eIFiso4F and eIFiso4F∙eIF4B
Source: PLoS One. 2024 May 2;19(5):e0300287. doi: 10.1371/journal.pone.0300287 (PMC11065315; doi:10.1371/journal.pone.0300287)
Supplement: S1 File — (ZIP) [file pone.0300287.s002.zip › Data supporting information files/S3 Data Fig 3.pdf]

Sample	0 time RLU	30 min RLU	60 min RLU
nonDWGE			17000
nonDWGE.VPg			86000
eIFiso4Fp+VPg	450	15000	34000
eIFiso4Fp.4B.VPg	399	17000	41000
eIFiso4Fp.PABP.VPg	500	19000	72000
eIFiso4Fp.4B.PABP.VPg	700	22000	77000
