# Supplementary material for: Poly(A)-binding protein promotes VPg-dependent translation of potyvirus through enhanced binding of phosphorylated eIFiso4F and eIFiso4F∙eIF4B
Source: PLoS One. 2024 May 2;19(5):e0300287. doi: 10.1371/journal.pone.0300287 (PMC11065315; doi:10.1371/journal.pone.0300287)
Supplement: S1 File — (ZIP) [file pone.0300287.s002.zip › Data supporting information files/S4 and S5 Data_Fig 4_5.pdf]

VPg nM	eIFiso4Fp	eIFiso4Fp.4	eIFiso4Fp.F	eIFiso4Fp.4	eIF4B	eIFiso4Fp.VPg71
0	0	0	0	0	0.00516	0.00363
25	0.17713	0.23495	0.30349	0.26473	0.02334	0.01981
50	0.28454	0.40212	0.47408	0.46557	0.04986	0.03713
75	0.38878	0.51366	0.58091	0.60687	0.06873	0.04934
100	0.46202	0.59831	0.67712	0.71052	0.08602	0.05814
150	0.55851	0.67509	0.76097	0.80663	0.09928	0.07092
200	0.65299	0.7295	0.8029	0.86617	0.11569	0.07887
250	0.70727	0.76518	0.821	0.89127	0.12836	0.08568
300	0.73714	0.79722	0.85976	0.90741	0.13475	0.08909
400	0.76442	0.81747	0.89308	0.93754	0.13662	0.08654
500	0.766	0.819	0.91778	0.95511	0.135	0.102
