# Supplementary material for: Poly(A)-binding protein promotes VPg-dependent translation of potyvirus through enhanced binding of phosphorylated eIFiso4F and eIFiso4F∙eIF4B
Source: PLoS One. 2024 May 2;19(5):e0300287. doi: 10.1371/journal.pone.0300287 (PMC11065315; doi:10.1371/journal.pone.0300287)
Supplement: S1 File — (ZIP) [file pone.0300287.s002.zip › Data supporting information files/S6 Data_Fig 6.pdf]

wavelength	eIFiso4Fp	eIFiso4Fp.VPg71	eIFiso4Fp.VPg	eIFiso4Fp.4B.VPg	eIFiso4Fp.PABP.VPg
260	0.49274	0.91602	-0.1519	1.3481	0.57739
259	0.42624	0.85027	-0.2259	1.2741	0.50181
258	0.49274	0.91602	-0.1615	1.3385	0.56227
257	0.50937	0.93246	-0.1404	1.3596	0.57739
256.01	0.54262	1.179	-0.31381	1.1862	0.60762
255	0.39299	1.2448	-0.25105	1.249	0.47158
254.01	0.49274	0.91602	-0.1569	1.3431	0.56227
253	0.29323	0.71877	-0.14345	1.3565	0.57739
252.01	0.16022	0.58727	-0.48715	1.0129	0.22975
251	0.09371	0.52152	-0.54692	0.95308	0.16929
250.01	-0.00605	0.42289	-0.87866	0.62134	0.03325
249	-0.0393	0.39002	-0.91004	0.58996	0.00302
248.01	-0.27207	0.15989	-1.1297	0.3703	-0.20859
247	-0.60459	-0.1853	-1.2552	0.2448	-0.3295
245.99	-0.6711	-0.25105	-1.5332	-0.0332	-0.42019
245	-0.82074	-0.39898	-1.6826	-0.1826	-0.76784
243.99	-1.1699	-0.74417	-2.0263	-0.5263	-0.91898
243	-1.2696	-0.8428	-2.3551	-0.8551	-1.2364
241.99	-1.8682	-1.4346	-2.7301	-1.2301	-1.6143
241	-2.0012	-1.5661	-3.0753	-1.5753	-1.7654
239.99	-2.2839	-1.8619	-3.3563	-1.8563	-2.0375
239	-2.7328	-2.3057	-3.8195	-2.3195	-2.3096
237.99	-3.4477	-3.0126	-4.1034	-2.6034	-2.7932
237	-3.9631	-3.5385	-4.6115	-3.1115	-3.1106
236	-4.9607	-4.3276	-5.1778	-3.6778	-3.6699
235	-5.9084	-5.2645	-6.1192	-4.6192	-4.214
234	-6.9226	-5.8562	-6.9277	-5.4277	-5.0302
233.01	-7.9535	-6.8918	-7.9588	-6.4588	-5.6348
232	-8.9178	-7.8452	-8.9121	-7.4121	-6.1941
231.01	-10.747	-8.6178	-9.8849	-8.3849	-6.9649
230	-12.343	-9.5711	-11.485	-9.985	-8.3706
229.01	-14.105	-12.398	-13.023	-11.523	-9.7158
228	-16.516	-15.242	-15	-13.5	-10.91
227.01	-18.694	-17.412	-17.824	-16.324	-12.364
226	-20.49	-19.417	-20.272	-18.772	-13.634
225.01	-22.651	-20.946	-22.657	-21.157	-14.828
224	-23.931	-22.014	-23.504	-22.004	-15.478
222.99	-24.497	-23.001	-23.63	-22.13	-16.007
222	-24.447	-23.806	-22.939	-21.439	-16.097
220.99	-24.563	-24.135	-22.186	-20.686	-15.931
220	-23.998	-24.003	-21.402	-19.902	-15.735
218.99	-23.615	-23.609	-20.366	-18.866	-15.493
218	-23.349	-23.346	-19.895	-18.395	-15.024
216.99	-23.266	-23.264	-19.801	-18.301	-14.722
216	-23.133	-22.918	-19.236	-17.736	-14.752
215	-23.499	-23.066	-19.174	-17.674	-14.692
214	-24.048	-23.625	-19.299	-17.799	-15.206
213	-24.713	-24.069	-19.519	-18.019	-15.432
212.01	-25.112	-24.677	-19.707	-18.207	-15.81

211	-26.109	-25.466	-20.272	-18.772	-16.188
210.01	-26.891	-26.042	-21.276	-19.776	-16.596
209	-27.473	-26.831	-22.061	-20.561	-16.989
208.01	-28.005	-27.143	-22.814	-21.314	-17.548
207	-28.537	-27.685	-23.128	-21.628	-17.669
206.01	-28.72	-27.439	-23.096	-21.596	-17.624
205	-27.622	-26.765	-22.657	-21.157	-16.793
204.01	-25.361	-24.94	-21.464	-19.964	-15.795
203	-21.62	-22.047	-19.456	-17.956	-14.586
201.99	-18.328	-18.119	-16.381	-14.881	-12.288
201.68	-14.504	-13.22	-12.772	-11.272	-9.8549
200.9	-10.896	-9.6204	-9.6025	-8.1025	-6.6959
200.58	-7.7207	-6.8589	-7.2803	-5.7803	-4.5647
200.25	-5.3597	-4.2947	-4.7071	-3.2071	-1.6022
199.94	-2.7161	-1.6482	-2.0711	-0.5711	0.84643
199.38	-0.05593	0.80096	0.8159	2.3159	3.5369
199.07	2.3881	3.4638	2.8243	4.3243	5.925
198.74	5.0151	6.0774	6.5272	8.0272	9.0387
198.43	8.224	8.8718	8.8807	10.381	11.79
198.1	10.452	10.877	11.548	13.048	14.268
197.78	13.179	13.606	14.278	15.778	16.793
197.45	15.29	15.496	17.04	18.54	19.362

eIFiso4Fp.4B.PABP.VPg
0.77388
0.69831
0.75877
0.77388
0.80411
0.66808
0.75877
0.97037
0.62273
0.56227
0.42624
0.39601
0.1844
0.06348
-0.02721
-0.37485
-0.526
-0.84341
-1.2213
-1.5689
-1.841
-2.1131
-2.5967
-2.9141
-3.2769
-3.821
-4.2443
-4.8488
-5.6046
-6.5719
-7.5846
-9.1264
-10.32
-11.771
-12.648
-13.253
-13.51
-13.449
-13.706
-13.54
-13.343
-13.102
-12.83
-12.724
-12.754
-12.693
-13.207
-13.434
-13.812

-14.19
-14.598
-14.991
-15.55
-15.671
-15.626
-14.794
-13.797
-12.588
-11.076
-9.2473
-7.0858
-4.7582
-2.1886
0.65296
3.3434
5.925
8.6487
11.4
13.274
15.012
16.584
