# Supplementary material for: Poly(A)-binding protein promotes VPg-dependent translation of potyvirus through enhanced binding of phosphorylated eIFiso4F and eIFiso4F∙eIF4B
Source: PLoS One. 2024 May 2;19(5):e0300287. doi: 10.1371/journal.pone.0300287 (PMC11065315; doi:10.1371/journal.pone.0300287)
Supplement: S1 File — (ZIP) [file pone.0300287.s002.zip › Data supporting information files/S7 Data_Fig 7.pdf]

1000/T (K-1)	lnKeq eIFiso4F.VPg	lnKeq eIFiso4Fp.VPg	lnKeq eIFiso4Fp.PABP
3.595	17.3	17.9	18.4
3.531	17	17.6	18
3.47	16.8	17.4	17.7
3.41	16.6	17.2	17.4
3.354	16.5	17	17.2

lnKeq eIFiso4Fp.4B.VPg	lnKeq eIFiso4Fp.4B.PABP.VPg
18	18.7
17.7	18.5
17.5	18
17.3	17.8
17.1	17.5
